# Introduction of neutralizing immunogenicity index to the rational design of MERS coronavirus subunit vaccines

**DOI:** 10.1038/ncomms13473

**Published:** 2016-11-22

**Authors:** Lanying Du, Wanbo Tai, Yang Yang, Guangyu Zhao, Qing Zhu, Shihui Sun, Chang Liu, Xinrong Tao, Chien-Te K. Tseng, Stanley Perlman, Shibo Jiang, Yusen Zhou, Fang Li

**Affiliations:** 1 Laboratory of Viral Immunology, Lindsley F. Kimball Research Institute, New York Blood Center, New York, New York 10065, USA; 2 State Key Laboratory of Pathogen and Biosecurity, Beijing Institute of Microbiology and Epidemiology, Beijing 100071, China; 3 Department of Pharmacology, University of Minnesota Medical School, Minneapolis, Minnesota 55455, USA; 4 Department of Microbiology and Immunology and Center for Biodefense and Emerging Disease, University of Texas Medical Branch, Galveston, Texas 77555, USA; 5 Department of Microbiology, University of Iowa, Iowa City, Iowa 52242, USA; 6 Key Laboratory of Medical Molecular Virology of Ministries of Education and Health, Shanghai Medical College and Institute of Medical Microbiology, Fudan University, Shanghai 200032, China

## Abstract

Viral subunit vaccines often contain immunodominant non-neutralizing epitopes that divert host immune responses. These epitopes should be eliminated in vaccine design, but there is no reliable method for evaluating an epitope's capacity to elicit neutralizing immune responses. Here we introduce a new concept ‘neutralizing immunogenicity index' (NII) to evaluate an epitope's neutralizing immunogenicity. To determine the NII, we mask the epitope with a glycan probe and then assess the epitope's contribution to the vaccine's overall neutralizing immunogenicity. As proof-of-concept, we measure the NII for different epitopes on an immunogen comprised of the receptor-binding domain from MERS coronavirus (MERS-CoV). Further, we design a variant form of this vaccine by masking an epitope that has a negative NII score. This engineered vaccine demonstrates significantly enhanced efficacy in protecting transgenic mice from lethal MERS-CoV challenge. Our study may guide the rational design of highly effective subunit vaccines to combat MERS-CoV and other life-threatening viruses.

## Abstract (web summary)

Viral subunit vaccines contain epitopes that elicit non-neutralizing immune responses. Here, Du *et al*. mask an immunodominant non-neutralizing epitope on a MERS coronavirus subunit vaccine with a glycan probe, leading to significantly improved efficacy of the vaccine.

## Graphical abstract

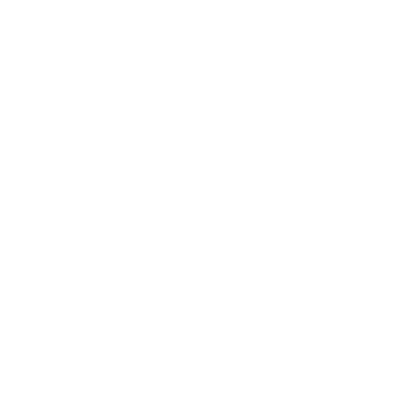

A major goal of viral subunit vaccine development is to rationally design immunogens that can elicit strong neutralizing immune responses in hosts^1^,^2^,^3^,^4^. The receptor-binding domains (RBDs) of virus surface spike proteins are the prime candidates for subunit vaccine design because they contain epitopes that can trigger strong immune responses^5^. In addition, viral RBDs play essential roles in viral infection cycles by binding to their host receptor for viral attachment^6^. Thus, part of the host immune responses elicited by viral RBDs can target the receptor-binding region and thereby neutralize viral entry into host cells. However, two problems potentially hinder the development of viral RBDs as subunit vaccines. First, viruses can evade the host immune responses elicited by their own spikes or RBD-based vaccines. One of the immune evasion mechanisms by viruses is to use immunodominant non-neutralizing epitopes on their RBDs to divert host immune responses, which has been thoroughly illustrated in the case of the HIV receptor-binding subunit gp120 (refs 1, 3). Second, when taken out of the context of the full-length spike proteins, recombinant viral RBD vaccines expose large areas of previously buried surfaces that likely contain immunodominant non-neutralizing epitopes. Whether an outcome of viral evolution or vaccine design, these immunodominant non-neutralizing epitopes on viral RBDs can outcompete other epitopes in triggering host immune responses, so that the resulting immune responses target these non-neutralizing epitopes while neglecting neutralizing epitopes on viral RBDs (refs 7, 8, 9, 10). Rational design of viral subunit vaccines aims to focus the immune responses on neutralizing epitopes through masking or deletion of immunodominant non-neutralizing epitopes^11^,^12^,^13^.

A critical gap in subunit vaccine design is the lack of an effective way to evaluate an epitope's neutralizing immunogenicity (that is, its capacity to elicit neutralizing immune responses). There have been extensive efforts to predict epitopes' immunogenicity based on the physical and chemical properties of the epitopes^14^. However, these methods are not designed to predict epitopes' ‘neutralizing' immunogenicity, which holds the key for subunit vaccine design. Although some experimental methods are available to measure the neutralizing immunogenicity of linear epitopes by taking linear peptides out of the context of proteins^15^,^16^, these methods do not work for conformational epitopes, which are prevalent on RBD-based viral vaccines^5^. Finding a way to measure the neutralizing immunogenicity of different conformational epitopes on viral RBDs will tremendously help rational design of viral subunit vaccines.

RBD-based coronavirus vaccines have been extensively pursued due to the threat that coronaviruses pose to human health. Coronaviruses are enveloped and positive-stranded RNA viruses. In 2002–2003, SARS coronavirus (SARS-CoV) infected over 8,000 people with ∼10% fatality rate^17^,^18^. Since 2012, MERS coronavirus (MERS-CoV) has infected about 1700 people with ∼36% fatality rate^19^,^20^. The RBDs from SARS-CoV and MERS-CoV both contain a core structure and a receptor-binding motif (RBM). Their core structures are highly similar, but their RBMs are markedly different^21^,^22^,^23^,^24^, leading to different receptor specificity: SARS-CoV recognizes angiotensin-converting enzyme 2 (ACE2), whereas MERS-CoV recognizes dipeptidyl peptidase 4 (DPP4)^6^,^25^,^26^. Both SARS-CoV and MERS-CoV RBDs are capable of eliciting strong neutralizing antibody responses^5^,^27^,^28^,^29^,^30^. On the one hand, because of the enriched neutralizing epitopes in their RBM and their high-yield expression as recombinant proteins, coronavirus RBDs are promising subunit vaccine candidates. Moreover, because of their relatively simple structures compared with the intact spike proteins, coronavirus RBDs provide an excellent model system for structure-based subunit vaccine design. On the other hand, recently determined cryo-EM structures of coronavirus spike proteins revealed that whereas the RBM of coronavirus RBDs is accessible, large surface areas of the RBD core structure are buried in the full-length spike proteins (Supplementary Fig. 1)^31^,^32^. Thus, when these previously buried areas on the surface of the RBD core become exposed in recombinant RBD vaccines, they likely contain immunodominant non-neutralizing epitopes that divert host immune responses. Therefore, coronavirus RBDs both hold promises and present challenges for vaccine development. It is critical to evaluate the neutralizing immunogenicity of different epitopes on coronavirus RBDs, such that immunodominant neutralizing and non-neutralizing epitopes can be preserved and eliminated, respectively.

In this study we introduce a novel concept ‘neutralizing immunogenicity index' (NII) to evaluate the neutralizing immunogenicity of different epitopes on viral subunit vaccines. As proof-of-concept, we used NII as a tool to identify epitopes with different neutralizing immunogenicity on a MERS-CoV-RBD-based vaccine. Furthermore, we successfully applied this tool and significantly enhanced the efficacy of the MERS-CoV RBD vaccine in protecting human-DPP4-transgenic mice from lethal MERS-CoV challenge. Our study fills in a critical gap in subunit vaccine design, and can facilitate rational design of subunit vaccines against MERS-CoV and other life-threatening viruses.

## Results

### Introduction of glycan probes onto epitopes on MERS-CoV RBD

To evaluate the neutralizing immunogenicity of a specific epitope on viral RBD vaccines, we can either delete or mask the epitope and then measure the corresponding changes in the vaccine's capacity to elicit neutralizing immune responses. Alanine scanning of vaccine-surface residues likely leads to changes in the vaccine's overall immunogenicity that are too subtle to be measurable using currently available experimental methods, while deletion of a whole epitope may disturb the tertiary structure of the viral RBD. Instead, in this study we chose to mask the epitope of interest using a host-cell-derived glycan probe. This approach is effective and convenient because the glycan probe can impose steric interference for the access of antibodies and immune cells to the epitope, and also because the glycan probe is unlikely to interfere with the folding and solubility of the RBD. To place the glycan probe on an epitope, we introduced the N-linked glycosylation motif, asparagine-X-threonine (where X is any amino acid other than proline)^33^, onto different epitopes on viral RBD vaccines using site-directed mutagenesis.

**Figure 1:**
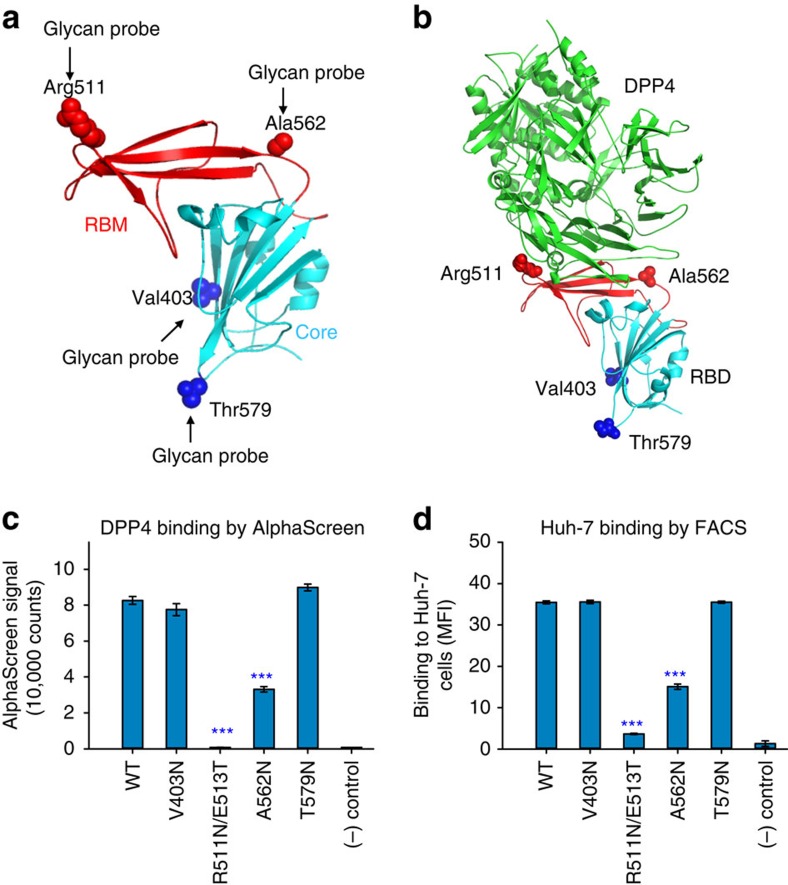
Introduction of glycan probes to MERS-CoV RBD vaccine. (**a**) Crystal structure of MERS-CoV RBD (PDB access code: 4L3N). The core structure is coloured in cyan, and the receptor-binding motif (RBM) in red. Four residues are shown where an N-linked glycan probe was introduced. (**b**) Structure of MERS-CoV RBD complexed with human DPP4 (PDB access code: 4KR0), showing the role of the four epitopes in the binding of the RBD to DPP4. (**c**) AlphaScreen assay was performed to detect the binding between recombinant MERS-CoV RBDs and recombinant human DPP4. PBS buffer was used as a negative control. Binding affinity was characterized as AlphaScreen counts. (**d**) Fluorescence-activated cell sorting (FACS) was carried out to detect the binding between recombinant MERS-CoV RBDs and cell-surface-expressed human DPP4. Human IgG protein was used as a negative control. Binding affinity was characterized as median fluorescence intensity. Error bars indicate s.e.m. ***: *P*<0.001.

As proof-of-concept, we chose to study several epitopes on the MERS-CoV RBD vaccine. The Fc-tagged RBD fragment containing residues from 377 to 588 was selected in this study because we previously showed that this fragment is a stable and effective vaccine candidate^34^. Four distinct epitopes on this MERS-CoV RBD fragment were selected based on their location on the RBD surface and their possible functional role in receptor binding: (i) Arg511 (located on a protruding loop and in the receptor-binding motif (RBM) region); (ii) Ala562 (located on a β-strand and in the RBM region); (iii) Val403 (located on a β-strand and in the core region); (iv) Thr579 (located on a protruding loop and in the core region) (Fig. 1a,b). On the basis of three-dimensional protrusion index map (Supplementary Fig. 2)^35^, the epitopes containing Arg511 and Thr579 both have a high protrusion index, whereas the epitopes containing Ala562 and Val403 both have a low protrusion index.

We introduced a glycan probe onto each of the above four epitopes on MERS-CoV RBD. To this end, we introduced single mutations V403N, T579N and A562N to pair with the already existent Thr405, Thr581 and Thr564, respectively, to generate three N-linked glycosylation sites. We also introduced double mutations R511N/E513T to generate the fourth N-linked glycosylation site. Each of these glycosylation sites was located in an individual MERS-CoV RBD fragment. We expressed and purified each of the four mutant RBDs in mammalian cells (Supplementary Fig. 3A).

### Characterization of RBDs containing engineered glycan probes

To test whether each of the above four epitopes on MERS-CoV RBD was actually glycosylated, we performed both SDS gel electrophoresis and mass spectrometry. Compared with the wild type RBD, each of the mutant RBDs exhibited a slower electrophoretic mobility on the gel, consistent with additional glycosylation (Supplementary Fig. 3A). Mass spectrometry revealed that the molecular weights of the mutant RBDs were ∼1–2 kDa larger than that of the wild type RBD, which was also consistent with an introduced glycan probe in each of the mutant RBDs (Supplementary Fig. 3B–F). For each of the purified mutant RBD samples, there was no visible presence of unglycosylated RBD on the SDS gel or the mass spectrometry spectrum (Supplementary Fig. 3). Thus, each of the four epitopes on MERS-CoV RBD had been successfully glycosylated.

To understand the correlation between the epitopes' role in receptor binding and their potential to be recognized by immune responses, we examined whether these engineered glycan probes on MERS-CoV RBD interfered with receptor binding. To this end, we used two alternative approaches. One approach was an AlphaScreen assay, which analysed the interaction between recombinant RBDs and recombinant human DPP4 in solution (Fig. 1c), and the other approach was fluorescence-activated cell sorting (FACS), which examined the interaction between recombinant RBDs and human DPP4 expressed on the Huh-7 cell-surface (Fig. 1d). The results from both assays revealed that the glycan probe located at residue 562 reduced the binding of the RBD to DPP4, the glycan probe located at residue 511 reduced the binding of the RBD to DPP4 even more, and the ones located at residues 403 and 579 had no impact on DPP4 binding. Structural analysis of the RBD/DPP4 interactions suggests that a glycan probe located at residue 511 would have serious steric clash with DPP4 binding, whereas a glycan probe located at residue 562 would have partial steric interference with DPP4 binding (Fig. 1b). Glycan probes located at residues 403 and 579 would be too far away from the receptor-binding region to have any impact on DPP4 binding. Hence, both the biochemical and structural analyses similarly elucidated the role of each of the glycan probes in the binding of the RBD to DPP4.

**Figure 2:**
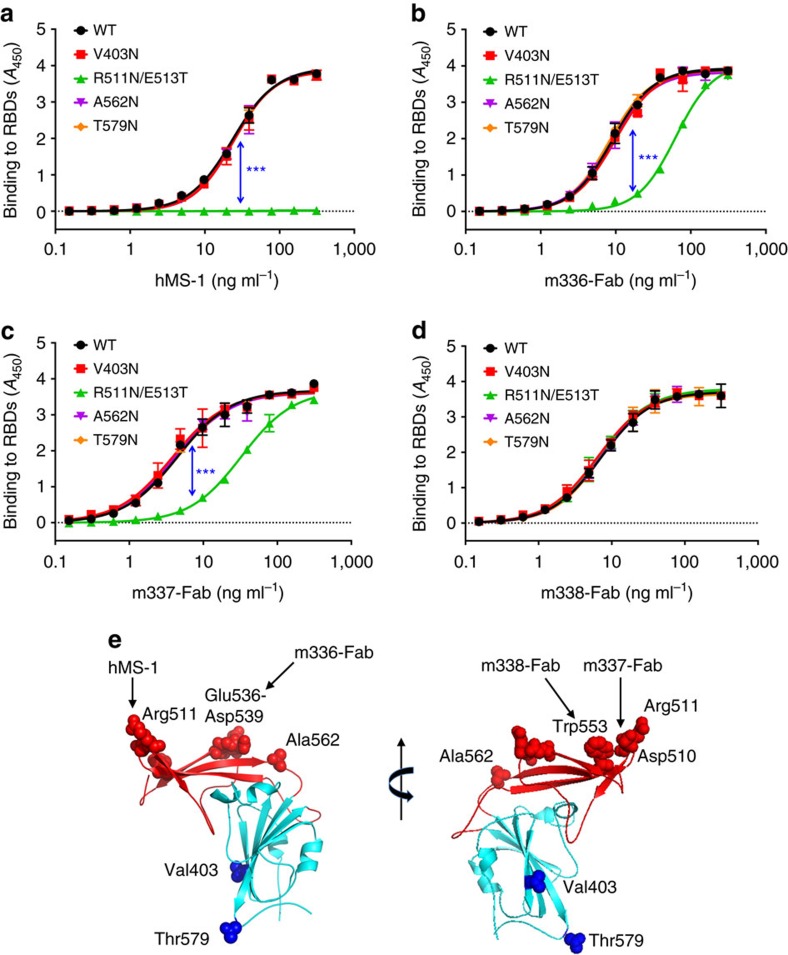
Role of engineered glycan probes in RBD binding to neutralizing mAbs. (**a**–**d**) ELISA was carried out to detect the binding between recombinant MERS-CoV RBD fragments and neutralizing mAbs. The binding affinity was characterized as the ELISA signal at 450 nm. Each of the mAbs was serially diluted before being used in ELISA. Error bars indicate s.e.m. ***: *P*<0.001. (**e**) Structure of MERS-CoV RBD, showing the identified binding site of the neutralizing mAbs on the RBD.

To understand the epitopes' potential to interact with neutralizing monoclonal antibodies (mAbs), we analysed how the engineered glycan probes interfered with the binding of the RBD to different neutralizing mAbs. We had access to four humanized mAbs (hMS-1, m336-Fab, m337-Fab, and m338-Fab). All of these mAbs were previously shown to be highly potent in neutralizing MERS-CoV infection of human cells^36^,^37^,^38^,^39^. ELISA between each of the RBDs and each of the mAbs demonstrated that the glycan probe located at residue 511 abolished the binding of the RBD to hMS-1 (Fig. 2a), reduced the binding of the RBD to m336-Fab and m337-Fab (Fig. 2b,c), and had no significant impact on the binding of the RBD to m338-Fab (Fig. 2d). In contrast, the glycan probes located at the other three residues, 403, 562 and 579, did not interfere with the binding of the RBD to any of the mAbs. The binding sites on the RBD for each of the mAbs were previously characterized through mutagenesis and/or structural studies^36^,^37^,^38^,^39^. Three of the four mAbs, hMS-1, m336-Fab and m337-Fab, bind at or near the epitope containing Arg511, whereas all of the mAbs bind away from the epitopes containing Ala562, Val403, and Thr579 (Fig. 2e). Overall, among the four selected epitopes, the epitope containing Arg511 played the most important role in the binding of neutralizing mAbs, and consequently the glycan probe covering this epitope interfered most with the binding of neutralizing mAbs.

This study thus far has characterized the structural features, receptor binding, and neutralizing mAb binding for four selected RBD epitopes using a glycan probe strategy. Each of the glycan probes introduced to one of the RBD epitopes only interfered with the binding of DPP4 or mAbs that interact with this specific epitope, but had no impact on the binding of DPP4 or mAbs to distant epitopes. This observation suggests that each of the glycan probes only shielded the epitope where the glycan probe was attached to, but did not affect the structures of other antigenic sites. It is consistent with findings obtained in studies on another viral spike protein, respiratory syncytial (RSV) virus F protein^40^.

### Measurement of neutralizing immunogenicity of RBD epitopes

**Figure 3:**
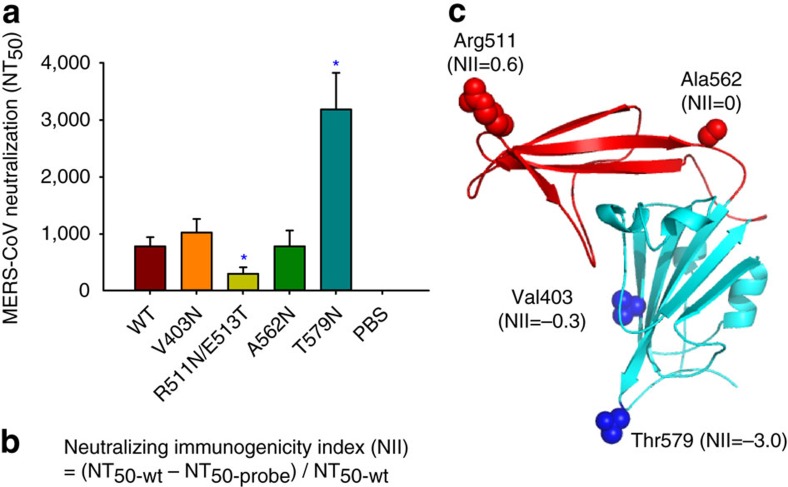
Measurement of neutralizing immunogenicity of RBD epitopes. (**a**) Measurement of neutralizing antibody titres of mouse sera induced by wild type (WT) or glycosylation mutant RBD. The neutralizing antibody titre of RBD-induced mouse sera was characterized by its capability to inhibit MERS-CoV-induced cytopathic effect (CPE) in cell culture. To this end, serially diluted mouse sera were added to MERS-CoV-infected cells, and the neutralizing antibody titre of the sera was expressed as the reciprocal of the highest dilution of sera that completely inhibited MERS-CoV-induced CPE in at least 50% of the wells (NT_50_) (Supplementary Table 1). PBS buffer was used as a negative control. Error bars indicate s.e.m. *: *P*<0.05. (**b**) Calculation of NII for each epitope. NT_50-wt_: NT_50_ for wild type RBD; NT_50-probe_: NT_50_ for RBD containing a glycan probe on one of the epitopes. (**c**) Mapping the calculated NIIs on the three-dimensional structure of MERS-CoV RBD.

To evaluate how the glycan probes altered the neutralizing immunogenicity (that is, the capacity to induce neutralizing immune responses) of MERS-CoV RBDs, we immunized BALB/c mice with each of the four RBDs containing one of the glycan probes. The immunization schedule is shown in Supplementary Fig. 4A. Sera were collected from mice immunized with each of the RBDs, and tested for MERS-CoV-neutralizing antibodies. Compared to the wild type RBD vaccine, the RBDs containing a glycan probe at residues 579 and 511 induced significantly higher and lower neutralizing antibody titres, respectively, in mouse sera, whereas the RBDs containing a glycan probe at residues 403 and 562 failed to induce significant changes in neutralizing antibody titres in mouse sera (Fig. 3a; Supplementary Table 1). Thus, masking the epitope containing Arg511 led to reduced neutralizing antibody titres in the immunized mice, demonstrating that this epitope made a positive contribution to the vaccine's overall neutralizing immunogenicity. Based on the same rationale, the epitope containing Thr579 made a negative contribution and the epitopes containing Val403 and Ala562 made insignificant contributions to the vaccine's overall neutralizing immunogenicity. The experiments were further repeated twice and similar results were obtained. These results provided a qualitative evaluation of the neutralizing immunogenicity for each of these epitopes.

How can we quantitatively evaluate the epitopes' neutralizing immunogenicity? Here we introduce a novel concept NII to describe an epitope's neutralizing immunogenicity. NII is defined as the contribution of an epitope to the vaccine's overall neutralizing immunogenicity. It can be determined by masking the epitope with a glycan probe and then measuring the relative change of the vaccine's overall capacity to elicit neutralizing antibody titres (Fig. 3b). Based on this definition, we calculated the NII for each of the four epitopes on the RBD (Fig. 3c; Supplementary Table 1). The epitope containing Thr579 had an NII of −3.0. The negative sign of the NII suggests a negative contribution from this epitope to the vaccine's overall neutralizing immunogenicity, and the value of the NII implicates that masking this epitope using a glycan probe increased the vaccine's overall neutralizing immunogenicity by three fold. Conversely, the epitope containing Arg511 had an NII of 0.6, suggesting that this epitope made a positive contribution to the vaccine's overall neutralizing immunogenicity and that masking this epitope using a glycan probe reduced the vaccine's overall neutralizing immunogenicity to 60% of that of the wild type vaccine. Therefore, the NII can serve as an effective tool to quantitatively evaluate the neutralizing immunogenicity of any epitope on the MERS-CoV RBD vaccine.

**Figure 4:**
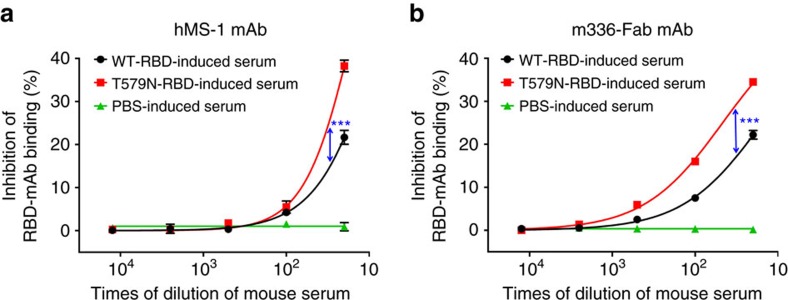
Masking negative epitope on the core led to immune refocusing on RBM. Competition assay was performed between neutralizing mAbs and glycosylation-mutant-RBD-induced mouse serum for the binding of wild type RBD. Specifically, ELISA was carried out between a neutralizing mAb, hMS-1 (**a**) or m336-Fab (**b**), and MERS-CoV RBD in the presence of mouse serum induced by the 579-glycosylated MERS-CoV RBD or mouse serum induced by the wild type MERS-CoV RBD. Mouse serum induced by PBS buffer was used as a negative control. Each of the sera was serially diluted before being used in the competition assay. For each serum dilution, the % reduction in mAb-RBD binding was computed for immune-sera present relative to immune-sera absent conditions. Error bars indicate s.e.m. ***: *P*<0.001.

To investigate why masking a negative epitope led to enhanced neutralizing immunogenicity of the MERS-CoV RBD vaccine, we performed a competition assay between neutralizing mAbs and mutant-RBD-induced mouse serum for the binding of wild type MERS-CoV RBD. More specifically, ELISA was carried out between a neutralizing mAb and MERS-CoV RBD in the presence of mouse serum induced by the 579-glycosylated MERS-CoV RBD (Fig. 4a,b). As a comparison, the mouse serum induced by the wild type MERS-CoV RBD was also included. Two different mAbs were used in the competition binding assay: hMs-1, which binds to the RBM epitope containing Arg511 (refs 36, 39), and m336-Fab, which binds to the RBM epitope surrounding Glu536-Asp539 (refs 37, 38). The result showed that the serum induced by the 579-glycosylated RBD inhibited the mAb-RBD binding significantly better than the serum induced by the wild type RBD, revealing enhanced neutralizing capability of the mouse serum due to the glycosylation at the 579 position. Moreover, the mouse serum induced by the 579-glycosylated RBD demonstrated enhanced binding for at least two separate neutralizing epitopes on the RBM, one surrounding Arg511 and the other Glu536-Asp539. Thus, masking an epitope on the RBD core structure with a high negative NII refocuses the host immune response on neutralizing epitopes on the RBM, leading to enhanced neutralizing immunogenicity of the RBD vaccine.

### Rational design of RBD vaccine with enhanced efficacy

To prove that highly effective MERS-CoV RBD vaccines can be rationally designed based on epitopes' neutralizing immunogenicity, we investigated the efficacy of two engineered MERS-CoV RBD vaccines using virus challenge studies. These engineered RBD vaccines have a negative epitope (that is, the epitope containing Thr579 and with an NII of −3.0) and a positive epitope (that is, the epitope containing Arg511 and with an NII of 0.6) masked, respectively, by a glycan probe. We chose to mask the epitopes rather than deleting them or mutating all of their residues to alanines because introducing a glycan is more convenient in practice and less disruptive to the immunogen's tertiary structure. The wild type RBD vaccine was used as a control. The animal model for vaccine testing was the lethal transgenic mouse model expressing human DPP4 (hDPP4-Tg mice)^41^,^42^. These mice were chosen for analysis because they are very susceptible to MERS-CoV and also because preventing disease in these mice is a stringent test of efficacy. The immunization schedule is shown in Supplementary Fig. 4B. Briefly, hDPP4-Tg mice were immunized with each of the RBD vaccines and challenged with MERS-CoV, and the survival rate and weight changes of the mice were recorded.

**Figure 5:**
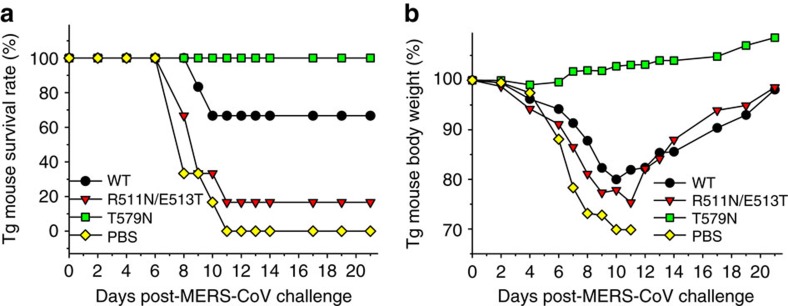
Rational design of MERS-CoV RBD vaccine with enhanced efficacy. Mice were immunized with two engineered RBD fragments containing a glycan probe at residue 511 (R511N/E513T) and residue 579 (T579N), respectively. Wild type RBD and PBS buffer were used as controls. Immunized mice were challenged with MERS-CoV (EMC-2012 strain), and observed for survival rate (**a**) and weight changes (**b**).

The efficacies of the RBD vaccines were evaluated based on the morbidity and mortality of the immunized and challenged mice. First, hDPP4-Tg mice immunized with the negative-epitope-masked RBD vaccine (that is, RBD containing T579N mutation) all survived MERS-CoV challenge (100% survival rate), whereas hDPP4-Tg mice immunized with the wild type RBD vaccine and with the positive-epitope-masked RBD vaccine (that is, RBD containing R511N/E513T mutations) demonstrated survival rates of 67 and 17%, respectively, after MERS-CoV challenge (Fig. 5a). Second, MERS-CoV challenge did not cause any weight loss in hDPP4-Tg mice immunized with the negative-epitope-masked RBD vaccine, but led to significant weight loss in hDPP4-Tg mice immunized with either the wild type RBD vaccine or the positive-epitope-masked RBD vaccine (Fig. 5b). The experiments were further repeated twice and similar results were obtained. These results revealed the enhanced efficacy of the negative-epitope-masked RBD vaccine and reduced efficacy of the positive-epitope-masked RBD vaccine, and demonstrated the utility of NII in developing a vaccine with increased immunogenicity in a stringent model of severe MERS.

## Discussion

Current vaccine design lacks an effective approach to evaluate the neutralizing immunogenicity of epitopes on viral subunit vaccines. In this study, we have developed a novel approach to measure vaccine epitopes' neutralizing immunogenicity. Using the MERS-CoV RBD as a model, we singly mask selected epitopes using host-derived glycan probes, and then measure the corresponding changes in the vaccine's overall neutralizing immunogenicity. We have also developed a method for calculating the NII for the selected epitopes. An epitope's neutralizing immunogenicity contains two parts: the neutralization capacity and immunogenicity. On the one hand, an epitope's neutralizing capacity is determined by the physical overlap of the epitope with the receptor-binding region and the potential role of the epitope in receptor binding. On the other hand, an epitope's immunogenicity is determined by its immune selfness (that is, how similar or dissimilar the viral epitope is to a host-originated epitope), protrusion, and other physical and chemical properties of the epitope. Logically, an epitope's NII is correlated with a combination of factors such as immune selfness, protrusion, potential overlap with receptor-binding region, and more. Because of the complex nature of NII, it is unlikely that the NII can be reliably predicted by software; instead, this study demonstrates that NII can be experimentally measured using the glycan probe approach.

As proof-of-concept, we measured the NII for four distinct epitopes on the MERS-CoV RBD vaccine, and also characterized the protrusion index, receptor binding, and monoclonal antibody binding of the RBDs each with an epitope masked by a glycan probe. The results revealed that the epitopes with a high and low protrusion index tend to have an NII with a high and low absolute value, respectively. In addition, epitopes within the receptor-binding region tend to have a positive NII, and the epitopes located outside the receptor-binding region tend to have a negative NII. We cannot correlate the immune selfness of epitopes with NII because there is no good method to evaluate the immune selfness of conformational epitopes. Overall, in rational design of viral subunit vaccines, the epitopes with a high positive NII should be preserved and exposed, while those with a high negative NII should be eliminated via deletion or masking. Indeed, our study has identified an epitope containing Thr579 as one with a high negative NII on MERS-CoV RBD. Thr579 is located on a protruding loop and away from the receptor-binding region, both of which contribute to its high negative NII. Importantly, Thr579 is buried inside the full-length coronavirus spike proteins, and only becomes exposed on the surface of the recombinant MERS-CoV RBD vaccine as an outcome of subunit vaccine design (Supplementary Fig. 1). To overcome this limitation of subunit vaccine design, the newly exposed epitopes with a high negative NII need to be masked or deleted.

To apply the NII strategy to vaccine design, we successfully enhanced the efficacy of the MERS-CoV RBD vaccine in virus challenge studies by masking its strong negative epitope (that is, the epitope containing Thr579, with an NII of −3.0) with a glycan probe. This engineered vaccine effectively protected hDPP4-transgenic mice from a lethal MERS-CoV infection. Compared with the wild type RBD vaccine, mice immunized with the engineered RBD vaccine showed increased neutralizing antibody responses in their sera; when challenged by MERS-CoV, they also demonstrated higher survival rate and less weight loss. These results prove that negative epitopes should be eliminated in vaccine design. In contrast, another engineered vaccine with a positive epitope masked (that is, the epitope containing Arg511, with an NII of 0.6) showed reduced efficacy in virus challenge studies, confirming that positive epitopes should be preserved and exposed in vaccine design. Taken altogether, we validated both the significance and feasibility of the NII strategy in vaccine design by successfully engineering a variant form of the MERS-CoV RBD vaccine with significantly enhanced efficacy.

Overall, our study contributes to viral subunit vaccine design in the following ways. First, our study introduces a new concept NII for the evaluation of how individual epitopes contribute to the overall neutralizing immunogenicity of subunit vaccines. Previous studies could not evaluate the neutralizing immunogenicity of conformational B-cell epitopes that dominate coronavirus RBD vaccines. Second, using the NII strategy our study identified an immunodominant non-neutralizing epitope on the surface of the MERS-CoV RBD core structure. This result shows that exposure of previously buried epitopes on viral subunit vaccines poses a challenge for subunit vaccine design. This concept, although needing further investigations, may be critical for the development of many viral RBD-based vaccines. Third, our study demonstrates that masking an immunodominant non-neutralizing epitope with a negative NII value on the surface of the MERS-CoV RBD core structure can shift host immune responses towards the neutralizing epitopes in the RBM region, providing means to overcome the limitation of viral subunit vaccines from vaccine design. Previous studies showed that hypervariable regions on HIV gp120 divert host immune responses and that masking these regions can shift host immune responses towards conserved neutralizing epitopes^11^,^12^, providing means to overcome the limitation of viral subunit vaccines from viral evolution. Fourth, although the NII strategy was used in the current study to improve the efficacy of viral subunit vaccines, it can also be potentially helpful in other epitope-based vaccine research. For example, previous studies masked or resurfaced non-neutralizing epitopes on viral immunogens, and used the engineered immunogens as baits to screen from neutralizing sera for monoclonal antibodies that bind to conserved neutralizing epitopes^43^,^44^,^45^,^46^. It is conceivable that the NII strategy can help identify immunodominant non-neutralizing epitopes on immunogens, allowing more targeted epitope modifications for efficient antibody screening. Finally, our study suggests that a three-dimensional ‘neutralizing immunogenicity map' (NIM) can be drawn to describe the distribution of epitopes with different neutralizing immunogenicity on the surface of viral subunit vaccines. Such an NIM can guide targeted masking of multiple strong negative epitopes, further enhancing the efficacy of viral subunit vaccines. We believe that our approach can facilitate the rational subunit vaccine design not only for coronaviruses such as MERS-CoV and SARS-CoV, but also for other life-threatening viruses such as HIV, influenza virus, and Ebola virus.

## Methods

### Animals

Female BALB/c mice of 6–8 week age mice and female human-DPP4-transgenic mice of 4-month age were used in the study. The animal studies were carried out in strict accordance with the recommendations in the Guide for the Care and Use of Laboratory Animals of the National Institutes of Health. The animal protocols were approved by the Committee on the Ethics of Animal Experiments of the New York Blood Center (Permit Number: 194.17) and Beijing Institute of Microbiology and Epidemiology (Permit Number: PMB15-0012).

### Cell lines

HEK293T (human embryonic kidney) and Vero E6 (monkey kidney) cells were obtained from American Type Culture Collection. Huh-7 (human hepatoma) cells were kindly provided by Dr Charles M. Rice at Rockefeller University. These cell lines were cultured in Dulbecco's modified Eagle medium (DMEM) supplemented with 10% fetal bovine serum (FBS), 2 mM L-glutamine, 100 units ml^−1^ penicillin, and 100 μg ml^−1^ streptomycin (Life Technologies Inc.). Sf9 insect cells were purchased from Life Technologies Inc., and cultured in Sf-900 III SFM medium supplemented with 100 units ml^−1^ penicillin and 100 μg ml^−1^ streptomycin (Life Technologies Inc.)

### Expression and purification of recombinant proteins

The expression and purification of recombinant MERS-CoV RBD was carried out as previously described^34^. Briefly, wild type (WT) RBD (residues 377–588; GenBank accession number: AFS88936.1) containing a C-terminal human IgG_1_ Fc tag was expressed in HEK293T cells, secreted into the cell culture supernatant, and purified by protein A affinity chromatography (GE Healthcare). Mutant RBD fragments containing engineered glycan probes were constructed via site-directed mutagenesis, and expressed and purified in the same way as the wild type RBD.

The expression and purification of recombinant human DPP4 was carried out as previously described^47^. Briefly, human DPP4 ectodomain (residues 39-766; GenBank accession no. NP_001926.2) containing an N-terminal human CD5 signal peptide and a C-terminal His_6_ tag was expressed in insect sf9 cells using the Bac-to-Bac expression system (Life Technologies Inc.), secreted to cell culture medium, and purified sequentially on HiTrap nickel chelating HP column and Superdex 200 gel filtration column (GE Healthcare).

### SDS gel electrophoresis

5 μg wild type or mutant MERS-CoV RBDs were subjected to SDS gel electrophoresis under denatured condition. Protein bands were stained using Coomassie Brilliant Blue R (Sigma-Aldrich), and image captured using myECL Imager (Life Technologies Inc.).

### Mass spectrometry

Wild type or mutant MERS-CoV RBDs at 100 μM concentration in 20 mM Tris-Cl, pH 7.4, 200 mM NaCl was ultrafiltrated with deionized water five times using an Amicon Ultra Centrifugal filter with a 10 kDa molecular weight cutoff. The desalted protein samples were subjected to MALDI-TOF Mass Spectrometry at Tufts University Core Facility. Mass spectrometry was performed in linear mode for molecular weight screening.

### AlphaScreen protein-protein binding assay

Binding between recombinant MERS-CoV RBDs and recombinant human DPP4 was measured using an AlphaScreen assay as previously described^34^,^36^. Briefly, 3 nM wild type or mutant MERS-CoV RBD with a C-terminal Fc tag was incubated with 300 nM human DPP4 with a C-terminal His_6_ tag at room temperature for 1 h. AlphaScreen protein A acceptor beads and nickel chelate donor beads (PerkinElmer Life Sciences) were added to the mixture at a final concentration of 5 μg ml^−1^ each. After incubation at room temperature for 1 h, the AlphaScreen signal was measured using an EnSpire plate reader (PerkinElmer Life Sciences), reflecting the binding affinity between the two proteins.

### FACS

The binding between recombinant MERS-CoV RBDs and human DPP4 expressed on the Huh-7 cell-surface was measured using fluorescence-activated cell sorting (FACS) as previously described^28^,^36^. Briefly, Huh-7 cells were incubated with wild type or mutant MERS-CoV RBD (1.25 μg ml^−1^) at room temperature for 30 min, followed by addition of FITC-conjugated anti-human-IgG-Fc polyclonal antibody (1:50 dilution) (Sigma-Aldrich) for 30 min. The amounts of RBD-bound Huh-7 cells were measured using flow cytometry, and the binding affinity between RBD and cell-surface DPP4 was characterized as median fluorescence intensity.

### Animal immunization and sample collection

Animal immunization and sample collection were carried out as previously described^34^. Briefly, BALB/c mice were subcutaneously immunized with wild type or mutant MERS-CoV RBD (10 μg per mouse) in the presence of Montanide ISA51 adjuvant^34^,^48^. PBS plus Montanide ISA51 was included as a negative control. Immunized mice were boosted twice with the same immunogen and adjuvant at a 3-week interval, and sera were collected 10 days after the last immunization for detection of neutralizing antibodies.

### ELISA

The binding between recombinant MERS-CoV RBD and neutralizing mAbs was measured using ELISA as previously described^36^. Briefly, ELISA plates were pre-coated with the same amount of wild type or mutant RBD (1 μg ml^−1^) overnight at 4 °C. After blocking with 2% non-fat milk at 37 °C for 2 h, serially diluted mAbs were added to the plates and incubated at 37 °C for 1 h. After washes, the plates were incubated at 37 °C for 1 h with horseradish-peroxidase-conjugated anti-human-IgG-Fab polyclonal antibody (1:5,000 dilution) (Sigma-Aldrich). Enzymatic reaction was carried out using substrate 3,3′,5,5′-tetramethylbenzidine (Life Technologies Inc.) and stopped with 1 N H_2_SO_4_. Absorbance at 450 nm (*A*_450_) was measured using ELISA Plate Reader (Tecan Group Ltd.).

The competition between neutralizing mAbs and mutant-RBD-induced mouse serum for the binding of wild type MERS-CoV RBD was carried out using ELISA as described above, except that the binding between wild type RBD and the neutralizing mAb (hMS-1 or m336-Fab at 5 μg ml^−1^ concentration) was performed in the presence of serially diluted mouse serum (T579N-RBD-induced, wild-type-RBD-induced, or PBS-induced). The RBD-mAb binding was detected by addition of horseradish-peroxidase-conjugated anti-human-IgG-Fab polyclonal antibody (1:5,000 dilution) and subsequent enzymatic reaction.

### Live MERS-CoV neutralization assay

A micro-neutralization assay was carried out to test neutralizing antibodies against live MERS-CoV as previously described^36^. Briefly, serially diluted mouse sera were incubated at room temperature for 1 h with ∼100 infectious MERS-CoV virions (EMC-2012 strain), and were then incubated with Vero E6 cells at 37 °C for 72 h. The neutralizing capability of the mouse sera was measured by determining the presence or absence of virus-induced cytopathic effect (CPE). Neutralizing antibody titres were expressed as the reciprocal of the highest dilution of sera that completely inhibited virus-induced CPE in at least 50% of the wells (NT_50_).

### MERS-CoV challenge studies

MERS-CoV challenge studies were carried out using human-DPP4-transgenic mice as previously described^41^. Briefly, mice were intramuscularly immunized with wild type or mutant MERS-CoV RBD (5 μg per mouse) in the presence of aluminium adjuvant^49^, and boosted once 4 weeks after the initial immunization. 12 weeks after the second immunization, mice were challenged with MERS-CoV (EMC-2012 strain, 10^4^ TCID_50_), and observed for 21 days for detection of survival rate and weight changes.

### Statistical analyses

In Fig. 1c–d, comparisons between WT RBD and each of the mutant RBDs in their binding to recombinant DPP4 by AlphaScreen (Fig. 1c) or to cell-surface DPP4 by FACS (Fig. 1d) were done using two-tailed *t*-test (***, *P*<0.001; 3 measurements for each RBD in Figs 1c and 4 measurements for each RBD in Fig. 1d).

In Figs 2a–d, nonlinear regression was performed using a log(inhibitor) versus normalized response—variable slope model. *R*^2^ of curve fit is larger than 0.97 for all curves in Fig. 2a–d, except for the curve representing R511/E513 mutant RBD in Fig. 2a where *R*^2^ of curve fit is 0.194. Comparisons between WT RBD and each of the four mutant RBDs in their binding affinity to mAbs by ELISA were done using the extra sum-of-squares F test (***, *P*<0.001; 12 different dilutions of each mAb, 4 measurements at each dilution for each mAb).

In Fig. 3a, comparisons between WT RBD and each of the mutant RBDs in their capacity to induce neutralizing serum in mice were done using two-tailed *t*-test (*, *P*<0.05; 4 measurements for each RBD).

In Fig. 4, nonlinear regression was performed using a log(inhibitor) versus normalized response—variable slope model. *R*^2^ of curve fit is larger than 0.98 for all curves in Fig. 4. Comparisons between WT-RBD-induced serum and T579N-RBD-induced serum in their inhibition of RBD/mAb binding by ELISA were done using the extra sum-of-squares F test (***, *P*<0.001; 4 different dilutions of each serum, 4 measurements at each dilution for each serum).

All statistical analyses were performed using GraphPad Prism 6 software.

### Data availability

All relevant data are available from the authors.

## Additional information

**How to cite this article**: Du, L. *et al*. Introduction of neutralizing immunogenicity index to the rational design of MERS coronavirus subunit vaccines. *Nat. Commun.* **7**, 13473 doi: 10.1038/ncomms13473 (2016).

**Publisher's note**: Springer Nature remains neutral with regard to jurisdictional claims in published maps and institutional affiliations.

## Supplementary Material

Supplementary Information Supplementary Figures 1-4 and Supplementary Table 1

## References

[b1] Kwong P. D., Mascola J. R. & Nabel G. J. Rational design of vaccines to elicit broadly neutralizing antibodies to HIV-1. Cold Spring Harb. Perspect. Med. 1, a007278 (2011). 22229123 10.1101/cshperspect.a007278 PMC3234457

[b2] Dormitzer P. R., Ulmer J. B. & Rappuoli R. Structure-based antigen design: a strategy for next generation vaccines. Trends Biotechnol. 26, 659–667 (2008). 18977045 10.1016/j.tibtech.2008.08.002 PMC7114313

[b3] Kulp D. W. & Schief W. R. Advances in structure-based vaccine design. Curr. Opin. Virol. 3, 322–331 (2013). 23806515 10.1016/j.coviro.2013.05.010 PMC4102719

[b4] Dormitzer P. R., Grandi G. & Rappuoli R. Structural vaccinology starts to deliver. Nat. Rev. Microbiol. 10, 807–813 (2012). 23154260 10.1038/nrmicro2893

[b5] Du L. . The spike protein of SARS-CoV–a target for vaccine and therapeutic development. Nat. Rev. Microbiol. 7, 226–236 (2009). 19198616 10.1038/nrmicro2090 PMC2750777

[b6] Li F. Receptor recognition mechanisms of coronaviruses: a decade of structural studies. J. Virol. 89, 1954–1964 (2015). 25428871 10.1128/JVI.02615-14 PMC4338876

[b7] Weaver J. M. . Immunodominance of CD4 T cells to foreign antigens is peptide intrinsic and independent of molecular context: implications for vaccine design. J. Immunol. 181, 3039–3048 (2008). 18713974 10.4049/jimmunol.181.5.3039 PMC2814425

[b8] Akram A. & Inman R. D. Immunodominance: a pivotal principle in host response to viral infections. Clin. Immunol. 143, 99–115 (2012). 22391152 10.1016/j.clim.2012.01.015

[b9] Patera A. C., Graham C. M., Thomas D. B. & Smith C. A. Immunodominance with progenitor B cell diversity in the neutralizing antibody repertoire to influenza infection. Eur. J. Immunol. 25, 1803–1809 (1995). 7621857 10.1002/eji.1830250702

[b10] Ito H. O., Nakashima T., So T., Hirata M. & Inoue M. Immunodominance of conformation-dependent B-cell epitopes of protein antigens. Biochem. Biophys. Res. Commun. 308, 770–776 (2003). 12927785 10.1016/s0006-291x(03)01466-9

[b11] Garrity R. R. . Refocusing neutralizing antibody response by targeted dampening of an immunodominant epitope. J. Immunol. 159, 279–289 (1997). 9200464

[b12] Schiffner T. . Immune focusing and enhanced neutralization induced by HIV-1 gp140 chemical cross-linking. J. Virol. 87, 10163–10172 (2013). 23843636 10.1128/JVI.01161-13 PMC3754013

[b13] Lin G. & Nara P. L. Designing immunogens to elicit broadly neutralizing antibodies to the HIV-1 envelope glycoprotein. Curr. HIV Res. 5, 514–541 (2007). 18045109 10.2174/157016207782418489

[b14] De Groot A. S. & Moise L. Prediction of immunogenicity for therapeutic proteins: state of the art. Curr. Opin. Drug. Discov. Devel. 10, 332–340 (2007). 17554860

[b15] Kessler J. H. . BCR-ABL fusion regions as a source of multiple leukemia-specific CD8^+^ T-cell epitopes. Leukemia 20, 1738–1750 (2006). 16932347 10.1038/sj.leu.2404354

[b16] Shirai M. . CTL responses of HLA-A2.1-transgenic mice specific for hepatitis C viral peptides predict epitopes for CTL of humans carrying HLA-A2.1. J. Immunol. 154, 2733–2742 (1995). 7533182

[b17] Ksiazek T. G. . A novel coronavirus associated with severe acute respiratory syndrome. N. Engl. J. Med. 348, 1953–1966 (2003). 12690092 10.1056/NEJMoa030781

[b18] Peiris J. S. M. . Coronavirus as a possible cause of severe acute respiratory syndrome. Lancet 361, 1319–1325 (2003). 12711465 10.1016/S0140-6736(03)13077-2 PMC7112372

[b19] Zaki A. M., van Boheemen S., Bestebroer T. M., Osterhaus A. D. & Fouchier R. A. Isolation of a novel coronavirus from a man with pneumonia in Saudi Arabia. N. Engl. J. Med. 367, 1814–1820 (2012). 23075143 10.1056/NEJMoa1211721

[b20] de Groot R. J. . Middle East respiratory syndrome coronavirus (MERS-CoV): announcement of the Coronavirus Study Group. J. Virol. 87, 7790–7792 (2013). 23678167 10.1128/JVI.01244-13 PMC3700179

[b21] Wang N. . Structure of MERS-CoV spike receptor-binding domain complexed with human receptor DPP4. Cell. Res. 23, 986–993 (2013). 23835475 10.1038/cr.2013.92 PMC3731569

[b22] Lu G. . Molecular basis of binding between novel human coronavirus MERS-CoV and its receptor CD26. Nature 500, 227–231 (2013). 23831647 10.1038/nature12328 PMC7095341

[b23] Li F., Li W., Farzan M. & Harrison S. C. Structure of SARS coronavirus spike receptor-binding domain complexed with receptor. Science 309, 1864–1868 (2005). 16166518 10.1126/science.1116480

[b24] Chen Y. . Crystal structure of the receptor-binding domain from newly emerged Middle East respiratory syndrome coronavirus. J. Virol. 87, 10777–10783 (2013). 23903833 10.1128/JVI.01756-13 PMC3807420

[b25] Li W. . Angiotensin-converting enzyme 2 is a functional receptor for the SARS coronavirus. Nature 426, 450–454 (2003). 14647384 10.1038/nature02145 PMC7095016

[b26] Raj V. S. . Dipeptidyl peptidase 4 is a functional receptor for the emerging human coronavirus-EMC. Nature 495, 251–254 (2013). 23486063 10.1038/nature12005 PMC7095326

[b27] He Y., Lu H., Siddiqui P., Zhou Y. & Jiang S. Receptor-binding domain of severe acute respiratory syndrome coronavirus spike protein contains multiple conformation-dependent epitopes that induce highly potent neutralizing antibodies. J. Immunol. 174, 4908–4915 (2005). 15814718 10.4049/jimmunol.174.8.4908

[b28] Du L. . Identification of a receptor-binding domain in the S protein of the novel human coronavirus Middle East respiratory syndrome coronavirus as an essential target for vaccine development. J. Virol. 87, 9939–9942 (2013). 23824801 10.1128/JVI.01048-13 PMC3754113

[b29] Du L. . Receptor-binding domain of SARS-CoV spike protein induces long-term protective immunity in an animal model. Vaccine 25, 2832–2838 (2007). 17092615 10.1016/j.vaccine.2006.10.031 PMC7115660

[b30] Zhang N., Jiang S. & Du L. Current advancements and potential strategies in the development of MERS-CoV vaccines. Expert Rev. Vaccines 13, 761–774 (2014). 24766432 10.1586/14760584.2014.912134 PMC4241375

[b31] Kirchdoerfer R. N. . Pre-fusion structure of a human coronavirus spike protein. Nature 531, 118–121 (2016). 26935699 10.1038/nature17200 PMC4860016

[b32] Walls A. C. . Cryo-electron microscopy structure of a coronavirus spike glycoprotein trimer. Nature 531, 114–117 (2016). 26855426 10.1038/nature16988 PMC5018210

[b33] Kornfeld R. & Kornfeld S. Assembly of asparagine-linked oligosaccharides. Annu. Rev. Biochem. 54, 631–664 (1985). 3896128 10.1146/annurev.bi.54.070185.003215

[b34] Ma C. . Searching for an ideal vaccine candidate among different MERS coronavirus receptor-binding fragments-The importance of immunofocusing in subunit vaccine design. Vaccine 32, 6170–6176 (2014). 25240756 10.1016/j.vaccine.2014.08.086 PMC4194190

[b35] Pintar A., Carugo O. & Pongor S. CX, an algorithm that identifies protruding atoms in proteins. Bioinformatics 18, 980–984 (2002). 12117796 10.1093/bioinformatics/18.7.980

[b36] Du L. . A conformation-dependent neutralizing monoclonal antibody specifically targeting receptor-binding domain in Middle East respiratory syndrome coronavirus spike protein. J. Virol. 88, 7045–7053 (2014). 24719424 10.1128/JVI.00433-14 PMC4054355

[b37] Ying T. . Exceptionally potent neutralization of Middle East respiratory syndrome coronavirus by human monoclonal antibodies. J. Virol. 88, 7796–7805 (2014). 24789777 10.1128/JVI.00912-14 PMC4097770

[b38] Ying T. . Junctional and allele-specific residues are critical for MERS-CoV neutralization by an exceptionally potent germline-like antibody. Nat. Commun. 6, 8223 (2015). 26370782 10.1038/ncomms9223 PMC4571279

[b39] Qiu H. . Single-dose treatment with a humanized neutralizing antibody affords full protection of a human transgenic mouse model from lethal Middle East respiratory syndrome (MERS)-coronavirus infection. Antiviral. Res. 132, 141–148 (2016). 27312105 10.1016/j.antiviral.2016.06.003 PMC5109928

[b40] Ngwuta J. O. . Prefusion F-specific antibodies determine the magnitude of RSV neutralizing activity in human sera. Sci. Transl Med. 7, 309ra162 (2015). 10.1126/scitranslmed.aac4241 PMC4672383 26468324

[b41] Zhao G. . Multi-organ damage in human dipeptidyl peptidase 4 transgenic mice infected with Middle East respiratory syndrome-coronavirus. PLoS ONE 10, e0145561 (2015). 26701103 10.1371/journal.pone.0145561 PMC4689477

[b42] Tao X. . Characterization and demonstration of the value of a lethal mouse model of Middle East respiratory syndrome coronavirus infection and disease. J. Virol. 90, 57–67 (2015). 26446606 10.1128/JVI.02009-15 PMC4702581

[b43] Wu X. . Rational design of envelope identifies broadly neutralizing human monoclonal antibodies to HIV-1. Science 329, 856–861 (2010). 20616233 10.1126/science.1187659 PMC2965066

[b44] Infante Y. C., Pupo A. & Rojas G. A combinatorial mutagenesis approach for functional epitope mapping on phage-displayed target antigen: application to antibodies against epidermal growth factor. mAbs 6, 637–648 (2014). 24589624 10.4161/mabs.28395 PMC4011908

[b45] Rojas G., Tundidor Y. & Infante Y. C. High throughput functional epitope mapping: revisiting phage display platform to scan target antigen surface. mAbs 6, 1368–1376 (2014). 25484050 10.4161/mabs.36144 PMC4622681

[b46] Malito E. . Defining a protective epitope on factor H binding protein, a key meningococcal virulence factor and vaccine antigen. Proc. Natl Acad. Sci. USA 110, 3304–3309 (2013). 23396847 10.1073/pnas.1222845110 PMC3587270

[b47] Yang Y. . Receptor usage and cell entry of bat coronavirus HKU4 provide insight into bat-to-human transmission of MERS coronavirus. Proc. Natl Acad. Sci. USA 111, 12516–12521 (2014). 25114257 10.1073/pnas.1405889111 PMC4151778

[b48] Aucouturier J. . 720 and 51: a new generation of water in oil emulsions as adjuvants for human vaccines. Expert Rev. Vaccines 1, 111–118 (2002). 12908518 10.1586/14760584.1.1.111

[b49] Montomoli E. . Current adjuvants and new perspectives in vaccine formulation. Expert Rev. Vaccines 10, 1053–1061 (2011). 21806399 10.1586/erv.11.48

