# Supplementary material for: Introduction of neutralizing immunogenicity index to the rational design of MERS coronavirus subunit vaccines
Source: Nat Commun. 2016 Nov 22;7:13473. doi: 10.1038/ncomms13473 (PMC5121417; doi:10.1038/ncomms13473)
Supplement: Supplementary Information — Supplementary Figures 1-4 and Supplementary Table 1 [file ncomms13473-s1.pdf]

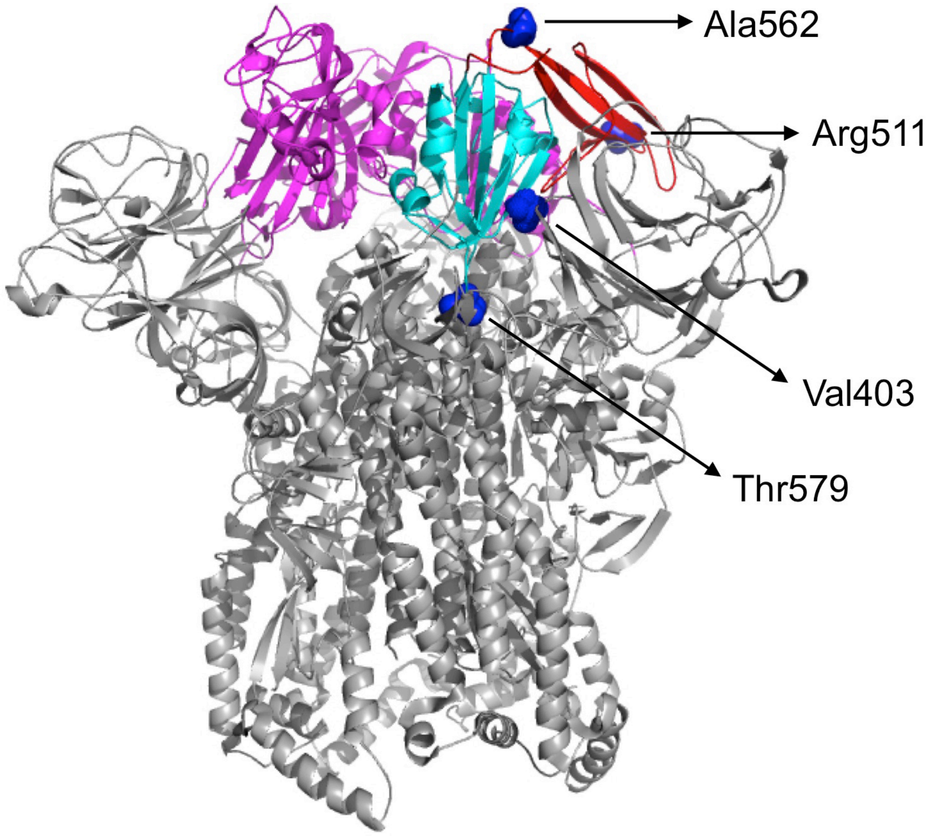

Supplementary Figure 1: The structure of MERS-CoV RBD modeled into the cryo-EM structure of trimeric spike protein of mouse hepatitis coronavirus (MHV). The modeling was based on the structural homology between MERS-CoV RBD (PDB access code: 4L3N) and the corresponding domain in the structure of trimeric MHV spike (PDB access code: 3JCL). The core structure of MERS-CoV RBD is in cyan, the RBM is in red, and the four selected residues for glycosylation on the surface on MERS-CoV RBD are in blue. There are three copies of the corresponding domain in the trimeric MHV spike protein, with two colored in magenta and the third replaced by MERS-CoV RBD.

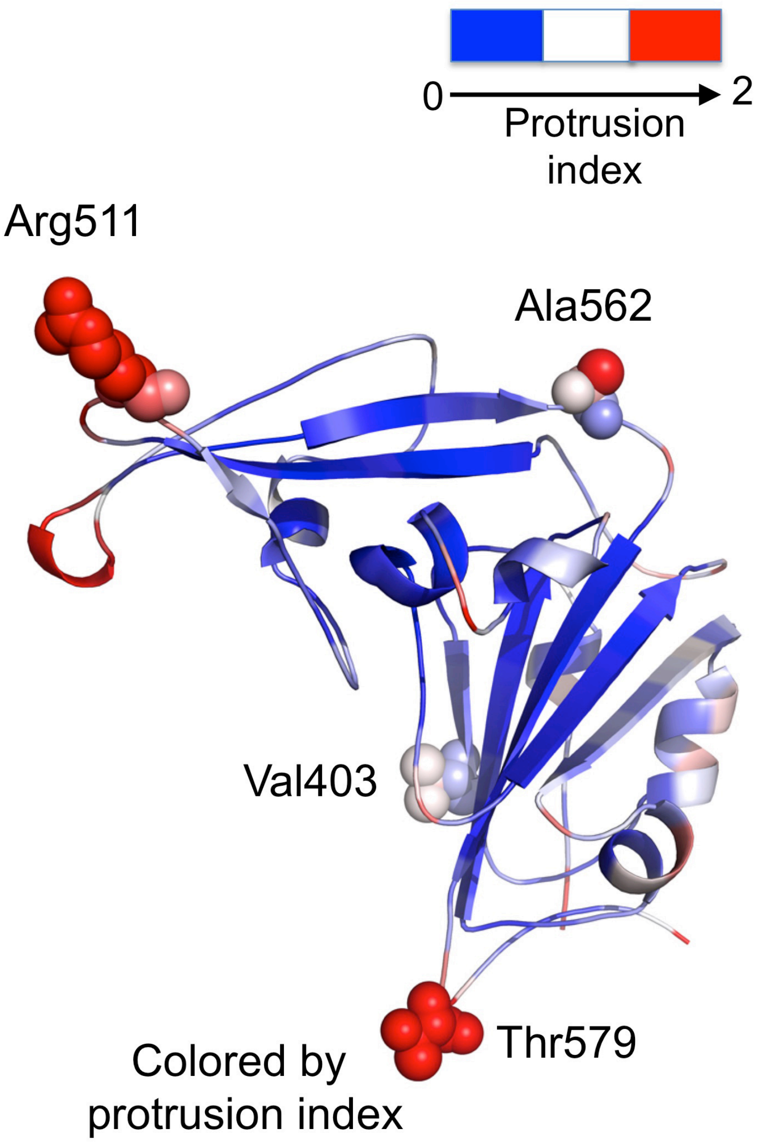

Supplementary Figure 2. Protrusion index map of MERS-CoV RBD. The protrusion index for each atom in the RBD was calculated. The structure is colored based on the protrusion index in the range of 0 (blue) to 2 (red).

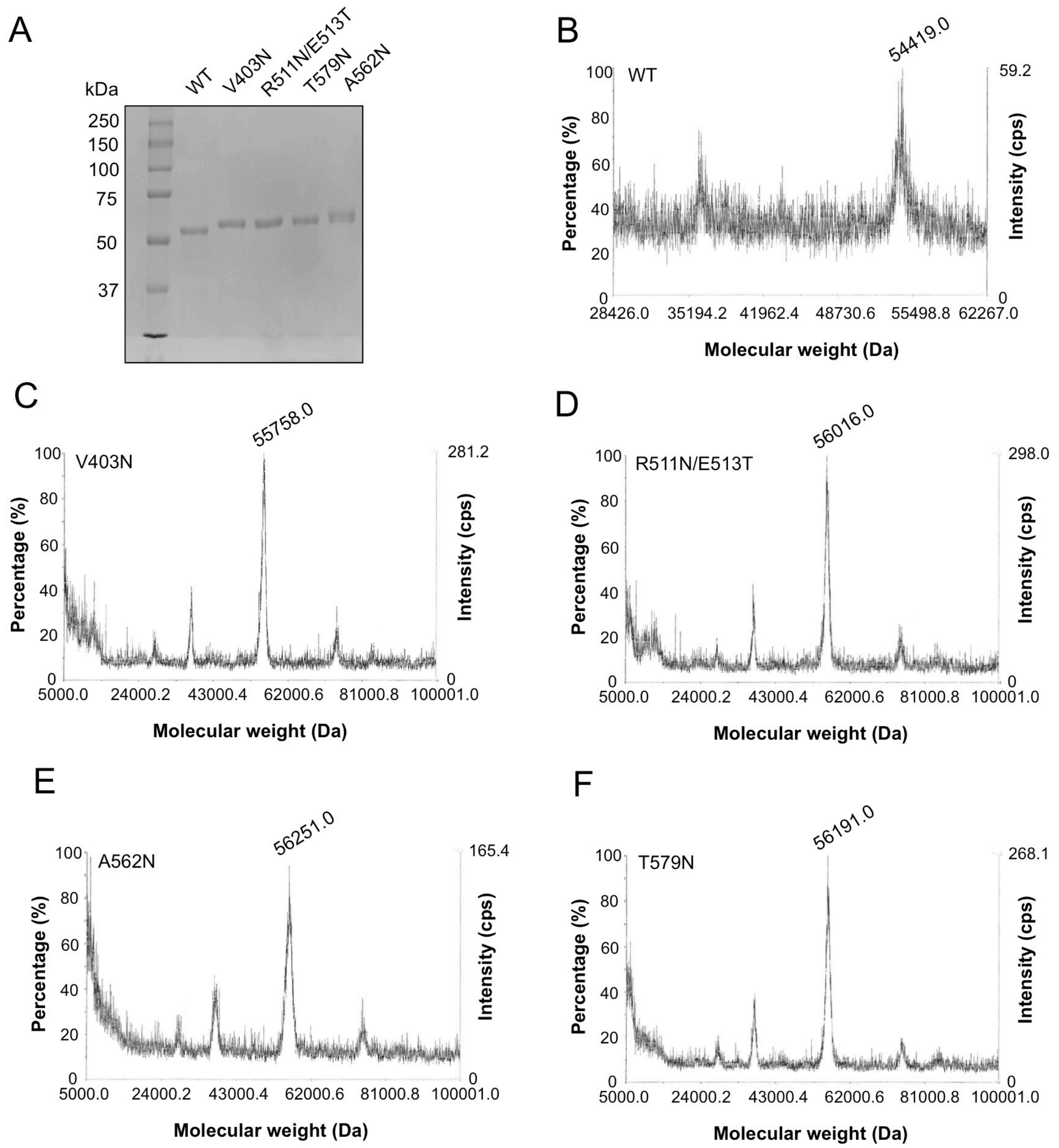

Supplementary Figure 3. Glycan probes have been successfully introduced onto MERS-CoV RBD vaccine. (A) SDS gel electrophoresis of wild type (WT) and glycosylation mutant MERS-CoV RBD fragments. (B)-(F) Mass spectrometry of wild type and mutant MERS-CoV RBDs. The results reveal a molecular weight increase of each of the mutant RBDs compared to the wild type RBD, confirming successful introduction of a glycan probe to the mutant RBDs.

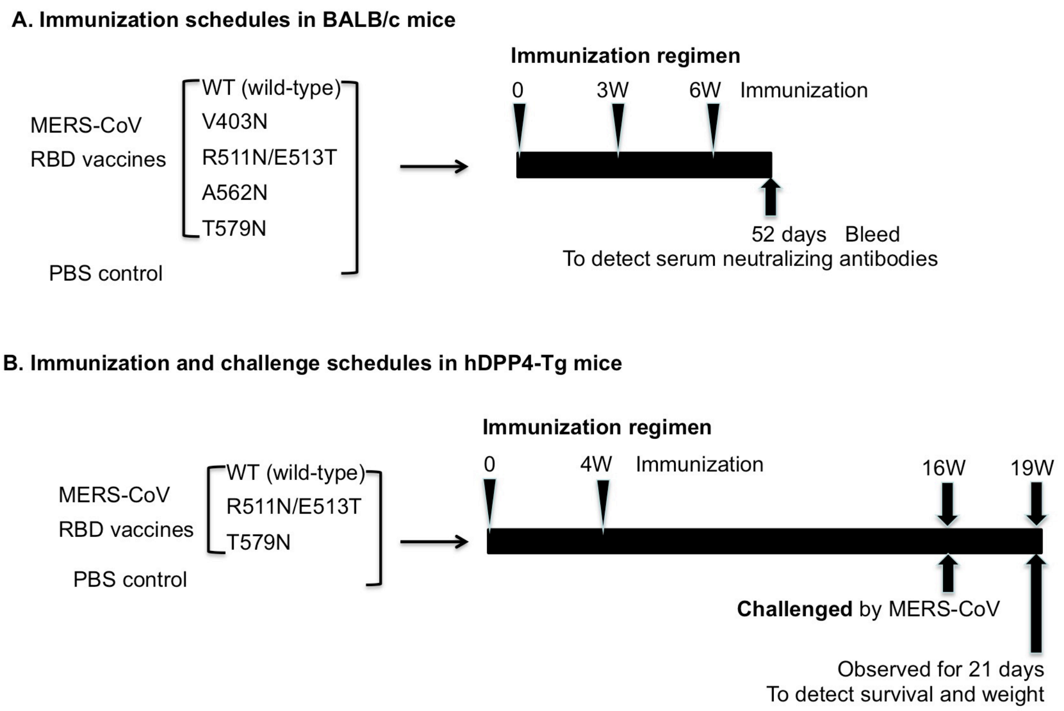

Supplementary Figure 4. Schedules for animal immunization, MERS-CoV challenge, and sample collection. (A) Immunization schedules in BALB/c mice. Groups of mice (N=4) were immunized with wild type or mutant MERS-CoV RBD. PBS was included as the control. Sera were collected for detection of neutralizing antibodies. (B) Immunization and MERS-CoV challenge schedules in human-DPP4-transgenic mice. Groups of mice (N=6) were immunized with wild type or mutant MERS-CoV RBD (R511N/E513T or T579N). PBS was included as the control. Immunized mice were further challenged with MERS-CoV, and observed for survival rate and weight changes.

Supplementary Table 1. MERS-CoV neutralizing antibody titers in RBD-immunized BALB/c mouse sera collected at 10 days after the last immunization

**RBD \ Group**	M1	M2	M3	M4	Mean	SEM	NII calculation
	**MERS-CoV neutralizing antibody titers (NT_{50})**						**$(NT_{50\text{-wt}} – NT_{50\text{-probe}}) / NT_{50\text{-wt}}$**
WT	640	1280	640	640	800	160	
V403N	1280	1280	1280	320	1040	240	(800-1040)/800 = -0.3
R511N/E513T	640	320	160	160	320	113	(800-320)/800 = 0.6
A562N	320	320	1280	1280	800	277	(800-800)/800 = 0
T579N	2560	5120	2560	2560	3200	640	(800-3200)/800 = -3.0

M1-M4: immunized mice in each group. Wild type RBD: WT. RBDs containing a glycan probe at the 403 (V403N), 511 (R511N/E513T), 562 (A562N), and 579 (T579N) positions, respectively. ND_{50}: the reciprocal of the highest dilution of sera that completely inhibited MERS-CoV-induced cytopathic effect (CPE) in at least 50% of the wells.
